# Aphid honeydew reduces soil nitrogen and increases host plant growth

**DOI:** 10.1007/s00442-026-05931-4

**Published:** 2026-07-15

**Authors:** Louie H. Yang, Lizbeth Gonzalez, Sarah Solís, Prabhjot Singh

**Affiliations:** Department of Entomology and Nematology, University of California, Davis, CA 95616 USA

**Keywords:** Plant-soil feedbacks, Plant-herbivore-soil feedbacks, Animal-soil effects, Immobilization, Milkweed-oleander aphids

## Abstract

Plants are known to affect their soils in ways that can have positive or negative effects on themselves and other plants, often described as plant-soil feedbacks. However, less is known about the potential for herbivores to change the soil in ways that affect plant growth. Many sap-feeding insect herbivores produce a sugary secretion called honeydew. Here, we ask if seasonal pulses of accumulated aphid (*Aphis nerii*) honeydew reduce the availability of soil nitrogen below milkweed (*Asclepias fascicularis*) host plants, and how honeydew-conditioned soil affects the relative seedling growth of host plants and other, potentially competing plant species. We hypothesized that: (1) honeydew would reduce plant-available nitrogen in the soil, reflecting the expectation for increased microbial immobilization in response to high carbon inputs, and (2) reduced nitrogen availability would have a greater negative effect on the growth of cheatgrass (*Bromus tectorum)* and yellow star-thistle (*Centaurea solstitialis*) than milkweed, reflecting the expectation of greater nitrogen limitation in disturbance-adapted, non-native, annual plant species compared with native, perennial plants. We found that the addition of honeydew at accumulated levels reduced total inorganic soil nitrogen by 48.8% relative to controls. Unexpectedly, milkweed seedlings were 90% taller and had 4.2 times greater dry biomass in honeydew-conditioned soil than in the control group, but we did not observe any effects on the growth of cheatgrass (*Bromus tectorum)* and yellow star-thistle *(Centaurea solstitialis*) seedlings. These results suggest the possibility of a positive plant-herbivore-soil feedback for milkweeds, mediated by aphid honeydew.

**Supplementary Information:**

The online version contains supplementary material available at 10.1007/s00442-026-05931-4.

## Introduction

The conceptual framework of plant-soil feedbacks describes how reciprocal interactions between plants and their soil can affect plant competition and coexistence (Bever 1994; Ehrenfeld et al. 2005; Van Der Putten et al. 2013). These interactions occur via wide-ranging mechanisms, including diverse physical, chemical and biotic processes (Ehrenfeld et al. 2005), but all plant-soil feedbacks fundamentally involve plants altering their soil in ways that affect subsequent plant performance (Bever 1994; Pernilla Brinkman et al. 2010). For example, plants can change their soil in ways that either improve their own performance (a positive direct feedback, e.g. via the accumulation of beneficial nutrients or microbes; Van Der Putten et al. 2013) or hinder it (a negative direct feedback, e.g. via autotoxicity or the accumulation of pathogens; Singh et al. 1999; Nijjer et al. 2007). In multispecies plant communities, the net effects of multiple feedbacks can be more complex or even counter-intuitive. For example, one plant species can have a positive net effect on itself even if its direct feedback effects are negative, as long as its soil modification has a sufficiently strong negative effect on the performance of a competing species (Bever et al. 1997).

Plants can alter the availability of soil nutrients in numerous ways, such as litter deposition and root exudation (Ehrenfeld et al. 2005). Whether plant- or herbivore-derived, the addition of organic matter with a high carbon-to-nitrogen (C: N) ratio (e.g. cellulose, lignin and honeydew) generally results in nitrogen immobilization (Janssen 1996; Wardle 2002; Ehrenfeld et al. 2005). Nitrogen immobilization is a fundamentally microbial process in which soil microbes compete with plants for nitrogen, effectively reducing plant-available nitrogen by incorporating it into soil microbial biomass (Schimel and Bennett 2004; Szili-Kovács et al. 2007; Cleveland and Liptzin 2007; Manzoni et al. 2008). This general expectation is supported in a wide range of systems for a wide range of organic inputs (e.g., Marion et al. 1982; Zink and Allen 1998; Perry et al. 2010), and is consistent with stoichiometric constraints (Cleveland and Liptzin 2007; Manzoni et al. 2008). While the effects of nitrogen immobilization are potentially complex in a multispecies plant community, several studies have shown that reducing soil nitrogen typically has a negative effect on nitrophilic plant species in a range of managed and unmanaged habitats (Zink and Allen 1998; Paschke et al. 2000; Blumenthal et al. 2003; Vasquez et al. 2008a; Perry et al. 2010; Gannett et al. 2024), and carbon addition has been tested as a means to reduce nitrogen availability and improve the relative performance of native plants in a restoration context, albeit often with mixed results (Paschke et al. 2000; Blumenthal et al. 2003; Corbin and D’Antonio 2004; Szili-Kovács et al. 2007; Vasquez et al. 2008b).

Honeydew-producing insect herbivores can have profound effects on nutrient cycling at the ecosystem level (Grier and Vogt 1990; Stadler and Michalzik 1998; Stadler et al. 2001), and the study of aphid honeydew contributes to an emerging understanding of how animals can affect biogeochemistry (Schmitz et al. 2014, 2018; Schmitz and Leroux 2020). Honeydew is a sugar-rich liquid produced primarily by phloem-feeding hemipteran insects, including aphids (Aphididae). At high densities, honeydew-producing insects can create a substantial flux of dissolved organic carbon from their host plants to the soil (Llewellyn et al. 1974; Seeger and Filser 2008). While some early studies proposed that honeydew could increase nitrogen availability by promoting nitrogen-fixing microbes in the soil (Owen and Wiegert 1976; Owen 1978), the consensus of later studies show that honeydew (and other high C: N inputs) generally reduce plant-available nitrogen in the soil due to the overwhelming effects of microbial immobilization (Buckley 1987; Grier and Vogt 1990; Stadler and Michalzik 1998; Wardle 2002; Michalzik and Stadler 2005; Milcu et al. 2015). Because terrestrial plant growth is commonly nitrogen-limited (Vitousek and Howarth 1991; LeBauer and Treseder 2008), honeydew addition would generally be expected to reduce plant growth (Grier and Vogt 1990). However, while the effects of honeydew on soil nitrogen have been studied at an ecosystem scale (Stadler and Michalzik 1998; Stadler et al. 2001; Milcu et al. 2015), few studies have examined honeydew as a potential mechanism of plant-soil (or plant-herbivore-soil) feedbacks. In the context of multispecies plant-soil feedbacks, a focal plant could potentially benefit from reducing nutrient availability in the soil if competing plants experience stronger negative effects than the focal plant.

**Fig. 1:**
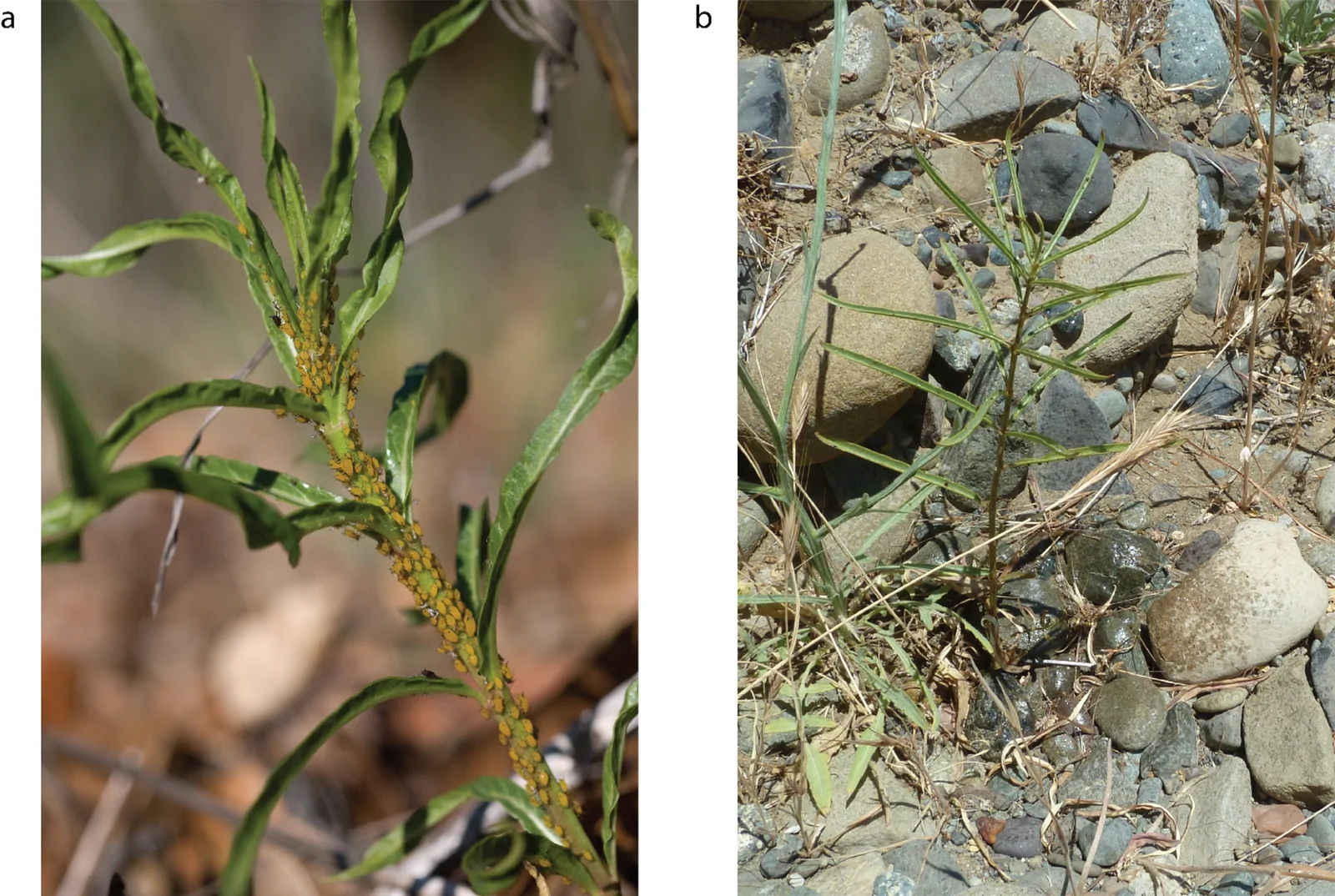
(**a**) Early-season narrow-leaved milkweed with milkweed-oleander aphids. (**b**) Late-season narrow-leaved milkweed with aphid honeydew glaze. Photos by L.H.Yang

The milkweed-oleander aphid, *Aphis nerii*, is a specialist herbivore of plants in the family Apocynacaeae (Groeters 1989). Although thought to be native to the Mediterranean, these bright yellow aphids are now widely distributed around the world (Harrison and Mondor 2011). Because they disperse widely and reproduce rapidly (Groeters 1989; Agrawal 2004; Ali and Agrawal 2014), they commonly achieve high densities on milkweeds (Price and Wilson 1979; Mooney et al. 2007; Mach et al. 2023), especially in warmer climates with mild winters (Fig. 1a, *pers. obs*). These aphids are only occasionally tended by ants (Bristow 1991; Smith et al. 2008), and ant-tending seems especially uncommon in xeric habitats (*pers. obs.*). This low level of ant-tending is possibly due to the high cardenolide concentration of the honeydew (Malcolm 1990; Pringle et al. 2014; Züst and Agrawal 2017). In an arid Mediterranean climate, the lack of summer rainfall and the low level of ant-tending means that the honeydew produced throughout the growing season commonly dries and accumulates on the soil surface below the host plant, sometimes forming a sugary glaze at the soil surface (Fig. 1b). This accumulated honeydew likely creates a pulsed input (*sensu* Yang et al. 2008) of labile, carbon-rich dissolved organic matter (32–35% sugars by dry weight, Pringle et al. 2014) in the soil following the first substantial rainfalls of the wet season. We ask the following questions: (1) Does this honeydew pulse drive nitrogen immobilization, decreasing inorganic nitrogen availability in the soil? (2) Does this honeydew pulse change the soil in ways that affect subsequent plant growth? and (3) Are the soil effects of honeydew different for the host plant species compared with other, potentially competing plant species? We hypothesized: (1) honeydew addition would reduce soil nitrogen availability, consistent with microbial immobilization, (2) this reduced soil nitrogen availability would reduce plant growth, consistent with nitrogen limitation, and (3) this reduced nitrogen availability would have stronger negative effects on potentially competing, nitrophilic plant species than on the host plant, creating the potential for net positive effects on the host plant.

## Methods

### Milkweed establishment

Narrow-leaved milkweed (*Asclepias fascicularis*) seeds (Hedgerow Farms, Winters, CA USA) were germinated in plug trays November 22, 2021 and January 4, 2022. These seedings were transplanted as 6–8 cm tall seedlings into 2.8 L plant containers filled with UC Davis Agronomy Mix soil (sand, redwood sawdust, pumice rock and sphagnum peat moss in a 1:1:1:1 ratio by volume, plus 2.4 kg m^− 3^ dolomite lime) between December 17, 2021 and February 26, 2022. These plants were grown to establishment (> 30 cm height) in a greenhouse at the UC Davis Orchard Park Research Facility before being moved outdoors. Plants were drip fertigated with a standard nutrient formula (see supplemental Table S1) throughout establishment. On June 21, 2022, aboveground growth was clipped to a height of approximately 30 cm to standardize aboveground biomass and encourage vigorous growth.

We selected 48 similar-sized milkweed plants and moved them into a 35.7 m^2^ temperature-controlled (16–30 °C) greenhouse on July 10, 2022. Twelve plants were placed on each of four greenhouse benches (i.e., experimental “blocks”) to account for variation within the greenhouse (see supplemental methods and analyses). These plants were initially irrigated without nitrogen for 12 days to reduce potential nutrient buildup, then returned to the standard fertigation regime for 38 more days. After this, irrigation was stopped to allow the soil to gradually dry down prior to the experimental honeydew manipulation.

### Aphids and honeydew

We introduced aphids on July 18, 2022 by placing cuttings with attached aphids from non-experimental donor plants on each experimental plant. Additional cuttings were added on July 20, July 21, and July 27, 2022 to promote rapid aphid colonization on all plants. Prior to the aphid introduction, water-resistant, vapor-permeable collars (14 cm diameter, DuPont Tyvek HomeWrap) were placed on the soil surface below each plant (but above the drip irrigation emitters) as a barrier to collect dried honeydew. These collars effectively prevented honeydew from entering the soil, mimicking the dry-season accumulation of honeydew observed in the field. We maintained these plants together with their aphid population for 63 days (until September 19, 2022) to approximate the magnitude of aphid honeydew accumulation observed in the field.

Our experimental soil manipulation was designed to simulate the pulsed addition of dissolved honeydew into the soil that would naturally occur in an arid Mediterranean climate following the first substantial rainfall of the wet winter season (i.e., as throughfall or stemflow, the incident precipitation that passes over plant surfaces to the soil, Parker 1983). On September 20, 2022, after 65 days of honeydew accumulation, we used 8 L of pure (reverse osmosis) water to dissolve the accumulated honeydew from the clipped aboveground biomass of all plants and their associated Tyvek collars. We randomly assigned each of the 48 soil-filled plant containers to receive one of three honeydew wash water concentrations (0%, 50% and 100%, *n* = 16 replicates per treatment, maintaining *n* = 4 replicates of each treatment on each bench) in a single pulsed input. The 100% treatment was scaled to reflect the approximate amount of honeydew accumulated on a single plant, dissolved in water, allowing for minor (10%) transport losses (i.e., all of the accumulated honeydew from 48 plants was dissolved in 8000 mL of water, resulting in 150 mL of honeydew wash water per plant allowing for 10% lost). Thus, the “0%” containers received 150 mL of pure water, the “50%” containers received 75 mL of honeydew wash water + 75 mL of pure water and the “100%” containers received 150 mL of honeydew wash water as a single pulsed input.

### Soil analysis

We conducted analyses to determine the concentrations of plant-available soil nitrogen in the spring following honeydew amendment. Ten days after the honeydew treatment (September 30, 2022), we collected shallow (< 10 cm) soil core samples from each container for nitrogen determination. We dried these samples at 40 °C and sieved them with a 1 mm mesh to remove roots and larger debris before submitting to the UC Davis Analytical Lab for ammonium and nitrate determination by flow injection analysis (Hofer 2003; Knepel 2003) on February 21, 2023. The ammonium determination was done on April 13, 2023 and the nitrate determination was done on April 4, 2023. We kept the remaining soil in the greenhouse until the plant performance bioassay, which began on April 7, 2023. Thus, the soil nitrogen analysis timeline approximated the timeline for the plant performance bioassay.

### Plant performance bioassay

We conducted a plant performance bioassay to quantify the effects of honeydew-conditioned soil on the growth of seedlings in the spring following honeydew amendment. On February 8, 2023, we homogenized the experimentally conditioned soil within each of the original 48 plant containers and distributed it into three smaller (approximately 550 mL) plant containers for separate bioassays of narrow-leaved milkweed, cheatgrass (*Bromus tectorum*) and yellow star-thistle (*Centaurea solstitialis*) seedling performance (144 containers total). Cheatgrass and yellow star-thistle are disturbance-adapted, non-native, annual species that are commonly thought to favor high-nitrogen soils (Larson and McInnis 1989; Lejeune and Seastedt 2001; Vasquez et al. 2008a; Blank and Morgan 2012; Stark and Norton 2015) and are potential competitors of milkweed. We planted seeds of each bioassay species directly into these 144 smaller containers on April 7, 2023. Cheatgrass and yellow star-thistle seeds were previously collected from local populations on July 8 and September 4, 2022, respectively. Additional narrow-leaved milkweed seeds were obtained from Hedgerow Farms (Winters, CA USA) on February 23, 2023. Planted containers were kept on a single bench in a temperature-controlled (16–30 °C) greenhouse and misted regularly with unfertilized water to maintain consistent soil moisture. We thinned seedlings to retain one focal seedling per container on April 14, 2023 and subsequently measured the height (i.e., maximum length) of surviving seedlings each week until May 19, 2023 (i.e., 42 days from planting) to approximate the duration of the early growth season prior to the dry summer season. We moved the plants to an outdoor bench without irrigation on May 26, and clipped the remaining shoot biomass of each plant at soil level on June 2, 2023, a total of 56 days after the start of this experiment. These samples were oven-dried at 40 °C and weighed.

### Statistical analysis

We considered three nested linear models of total inorganic nitrogen (i.e., the sum of ammonium and nitrate in ppm) including a numerical factor for treatment (0%, 50%, and 100%), a categorical factor for block and their interaction. The Akaike Information Criterion with a correction for sample size (AICc) (Bartoń 2025) favored the model including only the treatment factor, so we focused our analysis on this model. We confirmed the assumptions of residual normality and homoscedasticity using quantile-quantile and residual vs. fitted plots. We tested for the overall significance of the treatment factor using single-term deletion with a two-tailed *F*-test and estimated the proportion of observed variance explained by treatment (*η*^2^) as a measure of overall effect size.

For each bioassay species, we initially evaluated separate linear models of final plant biomass and height as a function of treatment, block and their interaction. We excluded plants that did not germinate from these analyses (11 of 48 milkweed, 14 of 48 cheatgrass, 1 of 48 star-thistle) but included plants that germinated and later died with biomasses and heights of zero (9 of 37 milkweed, 1 of 34 cheatgrass, 0 of 47 star-thistle). Because all AICc model comparisons favored the simplest model, our final models were constructed with treatment as the only explanatory factor. We confirmed the assumptions of residual normality and homoscedasticity in all models except for the analysis of milkweed biomass, which showed significant heterogeneity of variances. For this model, we applied a square root-transformation to meet the homoscedasticity assumption. In all models, we tested for the treatment effect using single-term deletion with a two-tailed *F*-test and used the proportion of observed variance explained by treatment (*η*^2^) to quantify the effect size.

All analyses were conducted in R (R Core Team 2025).

## Results

### Soil analysis

**Fig. 2:**
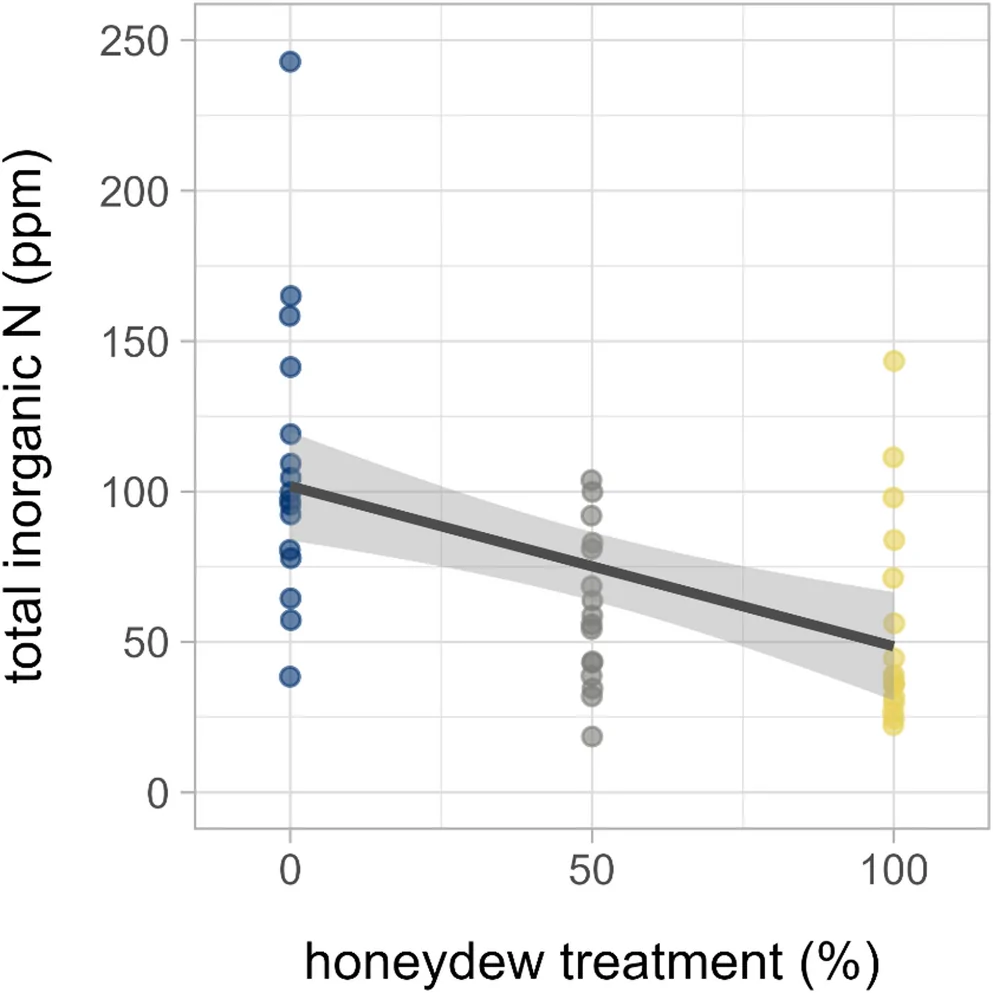
Total inorganic soil nitrogen by honeydew treatment

Aphid honeydew significantly decreased total inorganic nitrogen concentrations in the soil (Fig. 2, F_1,46_ = 14.7, *p* = 0.0004). Overall, the honeydew treatment explained 24% of the observed variation in total inorganic soil nitrogen concentrations (*η²* = 0.24). Compared to the control group (109 ppm), total inorganic N was 48.8% lower in the 100% honeydew treatment and 44.3% lower in the 50% honeydew treatment. When analyzed separately, both ammonium and nitrate also showed patterns consistent with soil nitrogen immobilization (ammonium: F_1,46_ = 7.1, *p* = 0.011; nitrate: F_1,46_ = 11.4, *p* = 0.001, supplemental Figure S1) similar to our analysis of total inorganic nitrogen.

### Plant performance bioassay

**Fig. 3:**
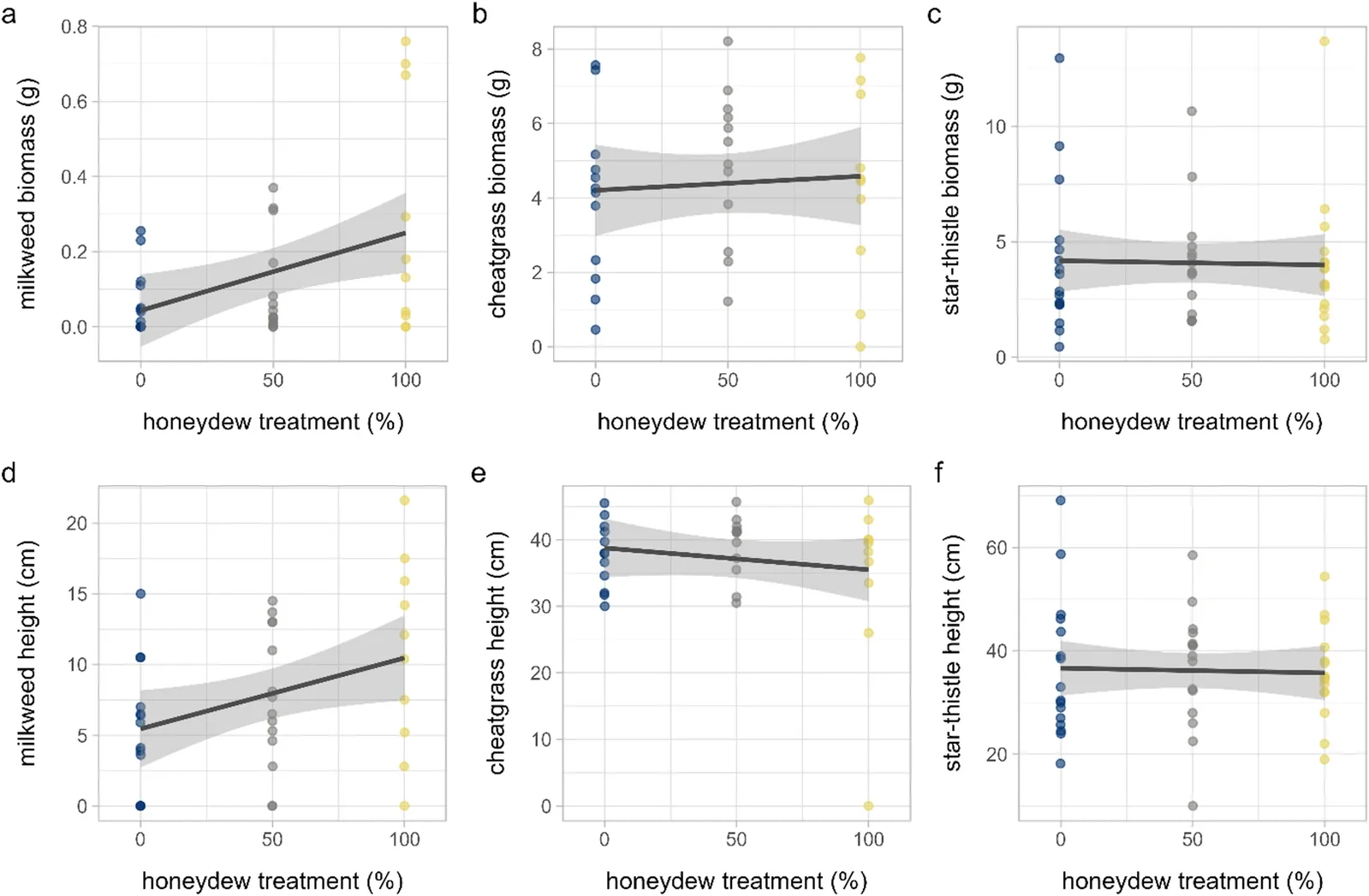
Aboveground plant biomass by treatment across three bioassay plant species: (**a**) narrow-leaved milkweed, (**b**) cheatgrass and (**c**) yellow star-thistle

Milkweed plants grew larger on honeydew-conditioned soil (Fig. 3a, biomass, F_1,35_=5.3, *p* = 0.027). The honeydew factor explained 14% of observed biomass variation (*η²* = 0.13), and the mean mass of plants in the 100% honeydew group was 4.2 times larger than those in the control group (0.28 vs. 0.067 g). The overall effect of treatment on plant height was similar (Fig. 3, F_1,35_=5.1, *p* = 0.030; *η²* = 0.13), and milkweeds in the 100% honeydew treatment grew 90% taller than control plants on average (see supplemental Figure S2, 10.7 vs. 5.6 cm). In contrast, we did not observe meaningful treatment effects on the biomass or height of cheatgrass (biomass, F_1,32_=0.15, *p* = 0.70; height, F_1,32_=0.86, *p* = 0.36) or yellow star-thistle (biomass, F_1,45_=0.03, *p* = 0.86; height, F_1,45_=0.05, *p* = 0.82).

## Discussion

The pulsed addition of aphid honeydew significantly reduced soil nitrogen availability in this experiment, consistent with our first hypothesis and the expectations of microbial stoichiometry (Janssen 1996; Cleveland and Liptzin 2007; Manzoni et al. 2008). These effects were large, with experimental honeydew amendments reducing soil nitrogen concentrations by nearly half compared with controls (Fig. 2). The magnitude and pulsed nature of the honeydew inputs in this system differ markedly from those studied in other systems (mostly mesic forests; Grier and Vogt 1990; Stadler and Michalzik 1998; Michalzik and Stadler 2005), but our soil nitrogen results are consistent in direction.

We hypothesized that this reduced soil nitrogen availability would have a negative effect on plant growth but instead saw *increased* growth (with both biomass and plant height metrics) in the milkweed host plant, and no effect on either metric for cheatgrass or yellow star-thistle. While we had anticipated that milkweeds might be more tolerant to reduced nitrogen availability than the other species in our study, the finding of increased growth in honeydew-conditioned soil was unexpected, and may suggest more complex nutrient, phytochemical, or microbial processes in the soil. Dissolved aphid honeydew is likely to be a substantial component of the throughfall and stemflow in this system, adding to an already complex mixture of chemical and microbial components. One possibility is that secondary metabolites in the honeydew (Malcolm 1990; Pringle et al. 2014; Züst and Agrawal 2017) directly or indirectly affected the growth of milkweed seedlings. Narrow-leaved milkweed has moderate constitutive and inducible concentrations of cardenolides compared with other temperate milkweed species (Agrawal and Fishbein 2006; Rasmann et al. 2009), though the cardenolides in *A. nerii* honeydew can differ from their host plant in both composition and concentration (Rothschild et al. 1970; Malcolm 1990; Pringle et al. 2014). While cardenolides have shown antimicrobial effects in other contexts (Akhtar et al. 1992; de Roode et al. 2008; Kumar et al. 2010; Kamtcha et al. 2018), little is known about their potential effects on soil microbes. It seems possible (but remains highly speculative) that milkweeds could have evolved over-compensatory responses to cardenolides in the soil, either due to their direct effects on the plant or their indirect effects mediated by changes in the soil microbial community.

The lack of negative effects on cheatgrass or yellow-star thistle was also unexpected, given that both species are known to have strong positive responses to increased soil nitrogen availability in establishment and competition (Larson and McInnis 1989; Lejeune and Seastedt 2001; Vasquez et al. 2008a). One possibility is that these species are less nitrogen-limited at the seedling stage, as most previous studies have focused on growth to maturity (Larson and McInnis 1989; Vasquez et al. 2008a). A study conducted by Monaco et al. (2003) is partially consistent with this possibility, as they also did not observe any effect of soil nitrogen on the short-term germination of individual cheatgrass seeds, though they did find positive fertilization effects in a longer hydroponic experiment. Another possibility is that some of the increased biomass observed in other studies reflects nitrogen-mediated changes in stand density rather than individual plant growth (Larson and McInnis 1989). A third (not mutually exclusive) explanation is that these species could have created their own positive plant-soil feedbacks. For example, cheatgrass has been shown to increase soil nitrogen availability in long-term plots, possibly via the production of lower C: N substrates (Stark and Norton 2015). It is unclear if this process could reduce their sensitivity of nitrogen immobilization on the shorter time scale of our experiment. Finally, a fourth speculative explanation is that the expected negative effects of nitrogen immobilization were counterbalanced by some unknown positive effect of the honeydew addition.

Although the specific direct effects of honeydew addition on seedling growth were unexpected, the pattern of relative effects was consistent with our third hypothesis: the effects of honeydew addition were more positive for the milkweed host plant than for the potentially competing plant species. This creates the potential for a positive plant-soil feedback effect mediated by aphid honeydew, even if the specific mechanisms of this effect remain unknown. In this way, this study builds on previous studies of honeydew effects at the ecosystem level to examine how honeydew-mediated changes in the soil might affect the host plant and other, potentially competing species (e.g., Milcu et al. 2015). Future studies will be necessary to investigate the robustness of (and potential mechanisms for) the observed growth response, and to further evaluate the role of herbivore inputs to the soil for plant growth and competition more generally.

Do milkweed responses to aphid honeydew constitute a plant-soil feedback? The reciprocal nature of plant-soil interactions is key to this conceptual model, and it may be unclear how the deposition of aphid honeydew fits into this framework. However, both aboveground and belowground herbivores have been shown to affect plant-soil interactions in multiple ways, including effects on plant-soil feedbacks (Wardle 2002; Van Der Putten et al. 2013 and references therein). If the excreta of a specialized herbivore can be understood as part of the plant’s extended phenotype, perhaps the key dynamic insights from this framework could apply as well. In any case, the study of plant-soil feedbacks already encompasses a wide diversity of systems and specific mechanisms (Ehrenfeld et al. 2005; Kulmatiski et al. 2008; Brinkman et al. 2010; Van Der Putten et al. 2013), and the further incorporation of herbivore-mediated effects into the explicit study of plant-herbivore-soil feedbacks suggests a potentially rich area for future investigation.

Our experiment did not aim to investigate the full complexity of the hypothesized mechanisms. While we infer that the addition of honeydew increased the microbial immobilization of soil nitrogen, we did not use field soil or directly measure changes in the microbial community. While we measured the performance of multiple potentially competing plant species, we did not directly test plants in a competitive context. While we focused on the important seedling establishment phase of three species (Shackelford et al. 2021), our experiment was both ontogenetically and taxonomically limited in scope. The honeydew input that characterizes this system is both localized and pulsed, and future studies should certainly consider longer timeframes, broader spatial extents and more species. On the other hand, the pulsed nature of this input is potentially characteristic of many other soil inputs (Kindlmann and Stadler 2004; Reynolds et al. 2004; Schaeffer and Evans 2005; James et al. 2006; Yang et al. 2008; Butterworth et al. 2023), especially in arid systems (Noy-Meir 1973; Reynolds et al. 2004; Feldman et al. 2018). The study of plant-soil feedbacks has long considered multiple temporal scales, including asynchronous or intermittent feedbacks (Ehrenfeld et al. 2005; Van Der Putten et al. 2013). While the effects of honeydew have primarily been studied in habitats with summer rainfall (e.g., Grier and Vogt 1990; Stadler et al. 1998; Seeger and Filser 2008; Milcu et al. 2015), these effects could be quite different in arid Mediterranean climates where honeydew dries and accumulates over the summer, then dissolves with the first rains of the wet season. In this context, our study was designed to examine the relatively short-term processes that result from this pulsed input of dissolved honeydew, and future studies will be necessary to examine the implications of this pulsed, seasonal input across multiple years.

This study contributes to our understanding of plant-herbivore-soil interactions. Previously, aphids in this system were known for their direct effects on host plant traits (Price and Wilson 1979; Ali and Agrawal 2014), and aboveground, plant-mediated indirect effects on other members of the milkweed-associated arthropod community (de Roode et al. 2011; Pringle et al. 2014; Ali and Agrawal 2014; Mach et al. 2023). However, our results indicate that aphids may also affect their host plants and the surrounding community through a belowground, honeydew-mediated pathway. More broadly, this work underscores the potentially important role of herbivore-soil interactions on community and ecosystem processes.

## Supplementary Information

Below is the link to the electronic supplementary material.

Supplementary Material 1

## Data Availability

Data are available on Dryad: 10.5061/dryad.mcvdnckcz.

## Code Availability

Data analysis scripts are available on Dryad: 10.5061/dryad.mcvdnckcz.
