# Supplementary material for: Aphid honeydew reduces soil nitrogen and increases host plant growth
Source: Oecologia. 2026 Jul 15;208(8):92. doi: 10.1007/s00442-026-05931-4 (PMC13373001; doi:10.1007/s00442-026-05931-4)
Supplement: Supplementary file 1 — Supplementary Material 1 [file 442_2026_5931_MOESM1_ESM.docx]

**Electronic Supplemental Material**

**Aphid honeydew reduces soil nitrogen and increases host plant growth**

Louie H. Yang^1, 2^, Lizbeth Gonzalez^1^, Sarah Solís^1^ and Prabhjot Singh^1^

^1^Department of Entomology and Nematology, University of California, Davis, CA 95616 USA

^2^*corresponding author*: lhyang@ucdavis.edu

*Oecologia*

**Table S1. Estimated standard fertigation nutrient concentrations**

|  | Standard (ppm) |
| --- | --- |
| NH_4_-N | 10 |
| NO_3_-N | 138 |
| Total-N | 150 |
| P | 50 |
| K | 200 |
| Ca | 175 |
| Mg | 55 |
| S | 120 |
| Fe | 2.5 |
| Cu | 0.02 |
| B | 0.50 |
| Mn | 0.50 |
| Mo | 0.01 |
| Zn | 0.05 |

**Supplemental methods and analyses**

*Milkweed establishment*

The research greenhouse used a climate control system that was designed to maintain internal greenhouse temperatures below 30° C using an evaporative cooling system. Ambient outdoor air was drawn through an evaporative cooling system on one side of the greenhouse as warmer internal air was exhausted from the other side. This system results in a temperature gradient within the greenhouse, with estimated daytime air temperatures near the evaporative cooling system around 23° C and air temperatures near the exhaust fans around 30° C during the experimental growing season (July 10 to September 19, 2022). The greenhouse benches were arranged linearly along this known temperature gradient, and the experimental design included benches as experimental blocks to account for this temperature variation.

*Soil analysis*

Our soil nitrogen analyses measured ammonium (NH_4_^+^) and nitrate (NO_3_^-^) concentrations, two measures that we summed into a single measure of total, plant-available, mineralized nitrogen. Both forms of nitrogen are typically present in soils, with the transformation of ammonium into nitrate mediated by nitrifying bacteria (Schimel and Bennett 2004). Plants use both and generally benefit from a combination of these two nitrogen ions (Raven and Smith 1976, Miller and Cramer 2005), as nitrate is reduced back to ammonium within the plant during the production of amino acids (Miller and Cramer 2005).

It is well-established that nitrifying bacteria are temperature-sensitive and that cooler soils generally show reduced nitrification rates (Frederick 1956; Sabey et al. 1956; Dai et al. 2020). As a result, the experimental blocks closest to the cooling system showed higher ammonium concentrations, with reduced ammonium (and correspondingly increased nitrate) concentrations in the warmer blocks. Our experimental blocks accounted for this temperature variation, but AICc analyses consistently favored models without this blocking factor. For the soil nitrogen analyses, this is because total plant-available nitrogen concentrations did not differ significantly between blocks, consistent the temperature-dependent conversion of ammonium into nitrate. AICc analyses of our plant performance experiment also favored models that excluded the block factor, indicating that observed differences in ammonium and nitrate did not significantly affect plant growth in our experiment. This lack of a block effect could indicate that ammonium and nitrate have similar growth effects in these bioassay species, or that initial differences in ammonium and nitrate concentrations attenuated quickly during the plant performance assay when the soils were maintained on a single warm germination bench that was automatically misted to maintain high soil moisture. Under these conditions, we estimate that initial differences in ammonium and nitrate concentrations would probably become undetectable in just a few days due to rapid nitrification (Frederick 1956; Sabey et al. 1956, 1959).

*Plant performance assay*

Germination was relatively quick and uniform in this study, and observed differences in plant size were unlikely to be attributable to differences in germination timing. When thinning, we intentionally retained the most centrally located seedling per container, to avoid bias by size or container edge effects. Germination success did not vary by treatment for any plant species (binomial GLM with treatment as the only explanatory factor; milkweed, χ^2^(1)=1.62, p=0.20; cheatgrass, χ^2^(1)=0.61, p=0.44; yellow star-thistle, χ^2^(1)=0, p=1). Similarly, survival to day 42 did not vary significantly by treatment (milkweed, χ^2^(1)=1.26, p=0.26; cheatgrass, χ^2^(1)=2.52, p=0.11; yellow star-thistle, χ^2^(1)=0, p=1).

**Figure S1.**

**
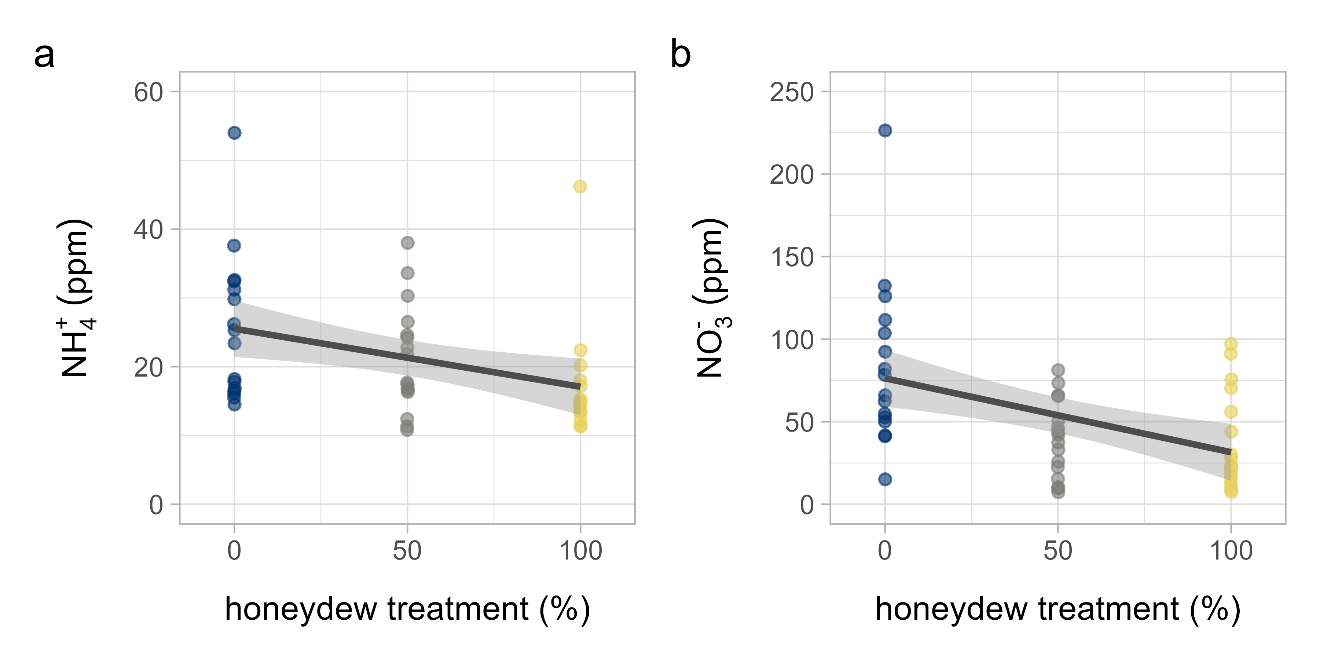
**

**Figure S2. Plant growth by treatment across three bioassay species**

**
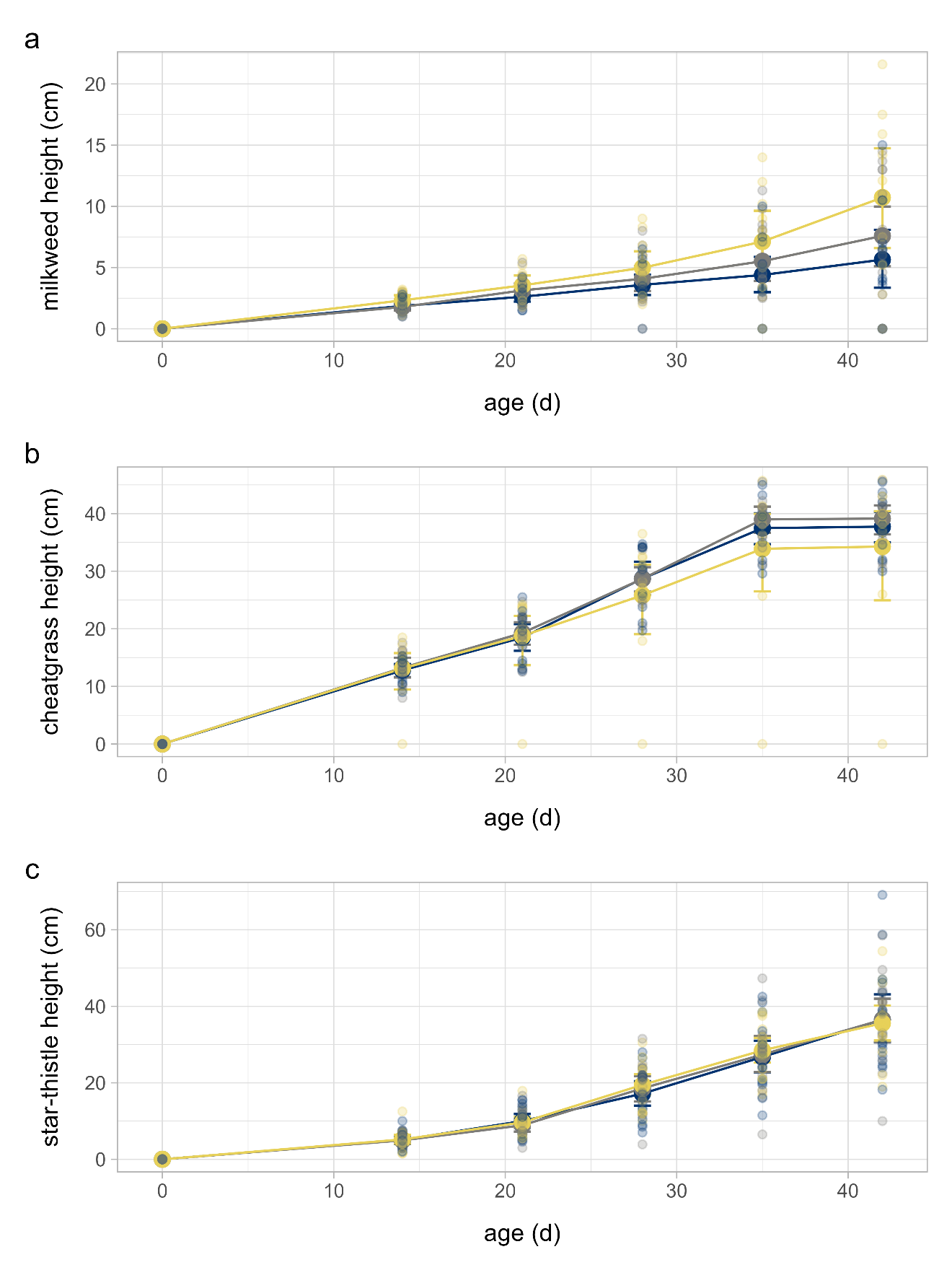
**
